# Development and application of consensus *in silico* models for advancing high-throughput toxicological predictions

**DOI:** 10.3389/fphar.2024.1307905

**Published:** 2024-01-25

**Authors:** Sean P. Collins, Brandon Mailloux, Sunil Kulkarni, Matthew Gagné, Alexandra S. Long, Tara S. Barton-Maclaren

**Affiliations:** Existing Substances Risk Assessment Bureau, Healthy Environments and Consumer Safety Branch, Health Canada, Ottawa, ON, Canada

**Keywords:** estrogen receptor, androgen receptor, endocrine disruption, genotoxicity, (Q)SAR, *in silico*, machine learning, consensus modeling

## Abstract

Computational toxicology models have been successfully implemented to prioritize and screen chemicals. There are numerous *in silico* (quantitative) structure–activity relationship ([Q]SAR) models for the prediction of a range of human-relevant toxicological endpoints, but for a given endpoint and chemical, not all predictions are identical due to differences in their training sets, algorithms, and methodology. This poses an issue for high-throughput screening of a large chemical inventory as it necessitates several models to cover diverse chemistries but will then generate data conflicts. To address this challenge, we developed a consensus modeling strategy to combine predictions obtained from different existing *in silico* (Q)SAR models into a single predictive value while also expanding chemical space coverage. This study developed consensus models for nine toxicological endpoints relating to estrogen receptor (ER) and androgen receptor (AR) interactions (i.e., binding, agonism, and antagonism) and genotoxicity (i.e., bacterial mutation, *in vitro* chromosomal aberration, and *in vivo* micronucleus). Consensus models were created by combining different (Q)SAR models using various weighting schemes. As a multi-objective optimization problem, there is no single best consensus model, and therefore, Pareto fronts were determined for each endpoint to identify the consensus models that optimize the multiple-criterion decisions simultaneously. Accordingly, this work presents sets of solutions for each endpoint that contain the optimal combination, regardless of the trade-off, with the results demonstrating that the consensus models improved both the predictive power and chemical space coverage. These solutions were further analyzed to find trends between the best consensus models and their components. Here, we demonstrate the development of a flexible and adaptable approach for *in silico* consensus modeling and its application across nine toxicological endpoints related to ER activity, AR activity, and genotoxicity. These consensus models are developed to be integrated into a larger multi-tier NAM-based framework to prioritize chemicals for further investigation and support the transition to a non-animal approach to risk assessment in Canada.

## 1 Introduction

In the evolving landscape of regulatory toxicology, significant effort is being taken to move away from toxicity testing using animals and toward the use of alternative methods, now commonly referred to as new approach methodologies (NAMs) (Andersen et al., 2010; Krewski et al., 2010; Richard et al., 2016). NAMs often refer to novel, non-animal, or alternative test methods, technologies, and/or innovative approaches developed to support chemical risk assessments (Harrill et al., 2019). Integrating NAMs into chemical assessment activities, especially in a multi-tiered framework, allows resources to be focused on high-priority substances and helps inform risk-based decisions that may otherwise be challenging due to a lack of data across a diverse chemical space. One notable example of ongoing efforts to advance the use of NAMs in regulatory toxicology is the development of integrated approaches to testing and assessment (IATA) under the Organization for Economic Co-operation and Development (OECD) through the Working Party on Hazard Assessment (OECD, 2020). Illustrative IATA case studies are submitted by member countries detailing the use of NAMs, including *in silico* models, to characterize chemical hazards. Another example of international science and regulatory communities supporting the progressive shift to the implementation of NAMs is Accelerating the Pace of Chemical Risk Assessment (APCRA) (Kavlock et al., 2018), which brings together governmental entities from around the world, including Health Canada (HC), the United States Environmental Protection Agency (USEPA), and the European Chemicals Agency (ECHA), to engage in the development of new hazards, exposure, and risk assessment methods aimed for use in regulatory chemical evaluation. As part of the APCRA, case studies are built using quantitative metrics derived from NAMs to inform prioritization and screening-level assessments, to continue to advance methods toward international acceptance for use in hazard identification and prediction, and to demonstrate the overall protection of human health and the environment from chemical exposures. A key component of novel testing and assessment strategies is the use of *in silico* tools such as machine learning (ML) or (quantitative) structure–activity relationships ([Q]SAR) models to support risk assessment activities. As the demand for non-animal test methods increases and new chemistries emerge, there exists a growing need to improve upon currently available (Q)SAR models and develop new *in silico* toxicity models to enhance the ability to predict across a broader chemical space and for additional effects of regulatory interest (Mansouri et al., 2016; National Institute of Technology and Evaluation, 2016; Madden et al., 2020; Collins and Barton-Maclaren, 2022).

Developing (Q)SAR models is not a new or unique endeavor, and it is common to have multiple available models for a single toxicological endpoint. For example, an activity of interest for endocrine disruption is estrogen receptor (ER) binding. ER binding has many commercial and public models available such as those developed by VEGA (Benfenati et al., 2013), ADMET (Simulations-Plus, 2023), ACD (Advanced Chemistry Development, 2019), CASE Ultra (CU) (Chakravarti and Saiakhov, 2022), the USEPA (Mansouri et al., 2016), and HC (Collins and Barton-Maclaren, 2022). With the wide availability of *in silico* models for each toxicological endpoint, the difficulty lies in determining the optimal model, or combination thereof, to apply in screening and weight-of-evidence assessment frameworks. Each model comprises specific attributes, including biases and errors, which can stem from the data used to train the model or its training processes, such as the type of models and descriptors applied. For example, using the substance 4,4′-dibromobenzophenone (CAS RN 3988-03-2) and evaluating ER-binding activity from six independent models, it was found that three models predicted the substance to be binding and three models predicted non-binding activity. This is not an uncommon result as when looking at the Collaborative Estrogen Receptor Activity Prediction Project (CERAPP) (Mansouri et al., 2016) evaluation dataset, only 88 of the 5,401 substances had complete concordance in predictions, and 161 had equal splits. Additionally, each model contains unique applicability domains (ADs), where it can predict the substance activity with confidence as only the substances within the AD are considered like the substances used to train the model. When working on a large chemical inventory across a diverse chemical space, such as the Canadian Domestic Substances List (DSL), multiple models are required for a given endpoint due to differences in ADs. However, the use of multiple models often results in discordant prediction results for a given chemical, and these data conflicts can cause confusion about which prediction should be selected for use in the decision-making context. This situation often makes model selection, transparency, and reproducibility difficult, especially if the predictions are integrated into automated screening approaches to be applied to thousands of chemicals in a high-throughput manner. Resolving model selection challenges and generating high-throughput predictions are important areas of research in Canada as the Government of Canada is required to generate a list of ministerial priority substances for assessment work as a part of the recent amendments to the Canadian Environmental Protection Act (CEPA) (Government of Canada, 2023).

One way to address the challenge of model selection and improve the predictive capabilities of the *in silico* models is to use consensus models, also known as ensemble models. A consensus model, as the name implies, combines the results of multiple models to provide a single outcome, thereby improving the performance. There are two primary advantages when using consensus models: smoothing out individual model errors and extending the AD. Consensus models have demonstrated utility for predictive toxicology and have been previously applied in efforts such as for CERAPP (Mansouri et al., 2016), Collaborative Modelling Project for Androgen Receptor Activity (CoMPARA) (Mansouri et al., 2020), Collaborative Acute Toxicity Modeling Suite (CATMoS) (Mansouri et al., 2021), and work at HC (Kulkarni and Barton-Maclaren, 2014) and others (Votano et al., 2004; Fang et al., 2015; Pradeep et al., 2016; Chauhan and Kumar, 2018; Zakharov et al., 2019; Ciallella et al., 2021; Schieferdecker et al., 2022). To create a consensus model, the predictions obtained from multiple *in silico* models (referred to as “component models”) are combined into a single prediction, which can be done through different combinatorial methods, such as a simple majority voting or an average of the results (Hewitt et al., 2007; Grisoni et al., 2019). More complex combinatorial methods can also be used, such as weighting the results using the model’s metrics or combinations of those metrics (Abdelaziz et al., 2016; Mansouri et al., 2016; Mansouri et al., 2020; Mansouri et al., 2021), or methodologies based on further combination practices (Pradeep et al., 2016; Zakharov et al., 2019; Valsecchi et al., 2020). The variety of different combinatorial methods (e.g., CERAPP weighting by the average of balanced accuracy on two sets, CoMPARA weighting by a score based on the goodness of fit, predictivity and robustness, or the approach applied by Pradeep et al. (2021) where a naive Bayes algorithm was used to train new ensemble models) shows that there is no defined procedure for combining models; therefore, it is practical to explore multiple combinatorial methodologies to search for the ideal method for a given application. This is typically not as simple as combining every available component model as previous works have demonstrated that this may not necessarily benefit the results (Kulkarni and Barton-Maclaren, 2014; Abdelaziz et al., 2016; Pradeep et al., 2021). It is possible that some models may provide “white noise” for the consensus model, and therefore, the results may be better if some models do not contribute. This creates a numerical problem as each method of combining results should be tested on all possible combinations of models for each toxicological endpoint.

This work aims to demonstrate a flexible and adaptable methodology for combining multiple *in silico* (Q)SAR models to yield a single optimal prediction for a toxicological endpoint using the Pareto front approach (Borghi et al., 2023; Casanova et al., 2023). It is important to note that the predictive power and chemical space coverage are distinct concepts that cannot be easily merged into a single metric. Often, when developing a consensus approach, a trade-off occurs when combining results (Shukla et al., 2012; De Buck et al., 2021). For example, predictive performance can be improved at the expense of chemical space coverage and *vice versa*. The Pareto front approach is an adaptable approach that allows one to identify the best performing consensus models across various metrics, regardless of trade-offs. In using this approach, we found the consensus models that optimize both a metric of predictivity and chemical space coverage. In this work, we demonstrate the development of a flexible and adaptable approach for *in silico* consensus modeling and its application across nine toxicological endpoints related to ER activity, AR activity, and genotoxicity. This is a NAM-based modeling approach that can be applied to large and/or diverse chemical inventories for high-throughput screening and prioritization.

## 2 Methods

### 2.1 Toxicological endpoints

The current study focuses on two broad toxicological categories: endocrine disruption and genotoxicity. The models examined for endocrine disruption activity cover three endpoints each for both ER and AR: binding, agonism, and antagonism. Genotoxicity also has three endpoints: bacterial mutation, *in vitro* chromosomal aberration, and *in vivo* micronucleus. These nine endpoints were chosen as they are well-defined toxicological endpoints with large high-quality datasets available. The datasets used to train the ER, AR, and genotoxicity models were obtained from the CERAPP (Mansouri et al., 2016), CoMPARA (Mansouri et al., 2020), and Leadscope databases (Leadscope, 2019), respectively. The datasets selected for this work were chosen due to their high-quality nature and the number of substances. If a dataset contains more substances, it is likely to cover a wider range of chemical space, leading to more robust models. It is important to note that some of the component models used in this work were trained on these datasets; however, it is considered that these datasets, due to their size and high-quality, represent the best choice for this work. Indeed, this is a difficult situation to avoid as model developers select chemicals from a variety of toxicological databases for training their respective endpoint specific models (Supplementary Table S1). The training sets tend to be a mix of publicly available data and proprietary data. As a result, not all the training set chemicals are accessible, especially in the commercial models. Across the different model training sets for a given endpoint, there is a high likelihood of the overlapping of chemicals. It is important to note that any new publicly available source of toxicological data is quickly used by model developers to retrain their existing models, unless the data are generated privately (Kulkarni and Barton-Maclaren, 2014). For the current study, such a truly external data source to validate our approach was not available; however, the consensus models are not fit to the data, but the data were used to validate the results. The methodology presented here can be easily transferred to other datasets if different data become available. Specific information related to the number of the component models and training datasets is given in Table 1. Further information about the datasets is given in Section 1, Supplementary Tables S1, S2.

**TABLE 1:** Information about consensus model development, including the number of component models, the dataset used to train the consensus models, and the number of substances in those datasets.

Endpoint	Component models	Training dataset	Number of substances
ER binding	6	CERAPP	5,401
ER agonism	5		6,318
ER antagonism	5		6,538
AR binding	5	CoMPARA	3,738
AR agonism	4		4,660
AR antagonism	4		3,882
Bacterial mutation	10	Leadscope	5,661
*In vitro* chromosomal aberration	8		1,203
*In vivo* micronucleus	6		927

### 2.2 *In silico* models

Consensus models require individual *in silico* models as their components. In total, 53 models were identified to cover the nine toxicological endpoints (Table 1); the number of models per endpoint ranged from 4 to 10 depending on the endpoint examined. These models came from various public and commercial sources such as HC-developed random forest models (Collins and Barton-Maclaren, 2022), the USEPA CERAPP (Mansouri et al., 2016) and CoMPARA (Mansouri et al., 2020) models, Advanced Chemistry Development (ACD) Percepta (Advanced Chemistry Development, 2019), VEGA (Satyanarayan et al., 2016), CU (Saiakhov et al., 2013), ADMET Predictor (Simulations-Plus, 2023), and Oasis TIMES (Todorov et al., 2011). The individual (Q)SAR models were chosen based on their availability and expert judgment, concerning their applicability to each endpoint. This judgment focused on selecting both free and commercially available models at our disposal and encompassing a mix of statistical, expert rule-based, and metabolism-based models. Consideration was given to prioritizing the predictive performance across diverse datasets and assessing the applicability domain. Interpretability was sought in the chosen models to gain a nuanced understanding of structure–activity relationships. The evaluation criteria included considerations of robustness, computational efficiency, and validation techniques, with a commitment to adhering to OECD QSAR validation principles for enhanced reliability. The quality and quantity of training data were scrutinized looking at the QSAR Model Reporting Formats (QMRFs) to ensure the representation of the relevant chemical space. Additionally, factors such as the ease of use, documentation, and support were considered. Balancing these criteria and aligning with OECD QSAR validation principles ensured the selection of robust and compliant (Q)SAR models tailored to specific needs and constraints.

The performance of each non-consensus model, hereafter referred to as the component models, is described in the Results section, along with that of the consensus models. The performance of the component models ranged in predictive power and coverage due to inherent differences in training sets and methodologies. Details about *in silico* models, including which ones were used for each endpoint and links to their QMRFs, are given in Supplementary Tables S1–S3.

### 2.3 Performance metrics

There are multiple metrics which can be used to describe *in silico* models; however, they can broadly be classified as quantifying either the coverage or predictive power of the model. Coverage is a term used to describe the number of substances within the AD of a given model. Coverage is expressed as a percentage and is calculated by taking the number of substances within the AD and dividing by the total number of tested substances. The AD describes the structural space around the chemical substances used to train an *in silico* model. Substances that are structurally similar to the substances used to train the model are likely within the AD. In contrast, substances that are structurally dissimilar to the training substances are interpreted as those outside the AD. For example, if only small organic molecules were used to train a model, using that model to make predictions on a metal alloy would be expected to result in an out-of-domain prediction. When a prediction is made on substances outside of an AD, the confidence in that prediction is reduced, and the result is most often discarded.

Second, the metric of predictive performance quantitatively describes how well a model can make a correct prediction. There is no single metric that can be used to establish the predictive performance; sensitivity (Sn), specificity (Sp), and balanced accuracy (BA) are typically used, as described in Eqs 1–3, respectively, and include the following terms or variables: true positive (TP), false negative (FN), true negative (TN), and false positive (FP). These are standard terms when the model predicts binary results (binary classification), with the resultant terms derived from the combination of observed and predicted values. An example of a confusion matrix illustrating the relationships between observed and predicted results and the binary classification model outcome terms is shown in Table 2. While sensitivity and specificity explicitly refer to how many positive or negative values, respectively, are correctly predicted, the BA is a more holistic term averaging the sensitivity and specificity. In addition to being holistic, the BA is also a term suited to dealing with imbalanced datasets, which is often the case for toxicological endpoints. Imbalanced datasets are ones where a class (e.g., positive or negative results) significantly outweighs the other class in a training set. As certain conventional metrics, such as accuracy, cannot appropriately handle imbalanced datasets, it is essential to apply metrics that can accommodate this to ensure a relevant comparison of the predictive performance:

$$Sn=\frac{TP}{TP+FN},$$ (1)

$$Sp=\frac{TN}{TN+FP},$$ (2)

$$BA=\frac{1}{2}\left(\mathrm{Sensitivity}+\mathrm{Specificity}\right).$$ (3)

**TABLE 2:** Confusion matrix showing the relationships between the observed and predicted results and outcomes of the binary classification model as TN, FP, FN, and TP.

		Predicted	
		Inactive	Active
Observed	Inactive	TN	FP
	Active	FN	TP

The predictive terms can also be more complex, as shown with the modified score_1_, predictivity, and modified predictivity (ModP), described in Eqs 4–6, respectively. The modified score_1_ is based on the score_1_ term from the CERAPP work (Mansouri et al., 2016) and was modified here to be used on a single dataset. Predictivity is a term from the CoMPARA work (Mansouri et al., 2020) aimed at having a high BA and similar specificity and sensitivity. Here, we adjusted this term, now called modified predictivity (ModP), where there is still interest in maintaining high BA; however, instead of using the absolute difference between sensitivity and specificity, only sensitivity is considered in the equation for ModP. This was done to favor the capture of active chemicals with a possible increase in false positives as the trade-off. This additional weighting on sensitivity was desired, given the method developed aimed at addressing an important need for high-throughput screening and prioritization of chemicals for further attention, while wanting to minimize the possibility of false negatives and accept the concomitant increase in false positives. Although any value can be chosen for the weighting of BA and sensitivity, we chose 0.7 and 0.3, respectively, to maintain the same weighting used in the predictivity from the CoMPARA work (Mansouri et al., 2020).

$$Modified\;score_{1}=BA*Coverage,$$ (4)

$$Predictivity=0.7*BA+0.3*\left(1-\left|Sn-Sp\right|\right),$$ (5)

Modified Predictivity=0.7*BA+0.3*Sn. (6)

### 2.4 Consensus model combinatorial methodology

Consensus models are based on combining multiple component models to yield a single prediction. This is typically done by collecting the predictions from each model and then using a mathematical formula to combine the results into a single outcome. For each possible combination of component models for the nine endpoints studied, six combinatorial methods were considered, namely, majority, four statistically weighted methods, and a k-nearest neighbor approach (kNN). In other words, for each unique combination of models (e.g., models A + B + C), the six above-mentioned methods for combining these models were used. The most straightforward combination is a majority prediction, where the mode is considered the overall prediction. For the four statistically weighted models, the individual component models were weighted on (a) BA, (b) modified score_1_, (c) predictivity, and (d) modified predictivity. The last combinatorial method used in this work was a kNN approach, which uses chemical space information to weight the component models. For each chemical, the average distance to the k-nearest neighbors is calculated, and more weight is given to predictions with a shorter average distance. Further details about the kNN process can be found in Supplementary Materials (Section 2).

Before applying the combinatorial methodology, we first used each individual model to make predictions on their respective datasets. From these results, we calculated various statistical values, including sensitivity, BA, and coverage on the training datasets. These statistics were then used to determine the weight for the four statistical weighting methods: BA, modified score_1_, predictivity, and modified predictivity (Eqs 3–6). Additionally, for each substance, we computed the mean distance to its 12 nearest neighbors, which is used in the kNN combinatorial method. These approaches, combined with a simple majority voting, yield a total of six combinatorial methods.

The initial step in our process involved determining whether or not a substance fell within the AD of the consensus model. To establish AD inclusion, we applied a “majority weighting” approach, requiring substances to fall within the AD of the majority of its component models, as determined by their respective weights. For instance, we consider a consensus model created using BA weighting of four component models with BAs of 0.8, 0.75, 0.65, and 0.6 (summed weight of 2.8). In this case, a substance needed to fall within the AD of enough models to achieve a total summed weight of at least 1.4. For example, if a substance was within the AD of the second and fourth models, but not of the other two models, it would have a summed weight of 1.35 (0.75 and 0.6, respectively) and be considered outside the AD of the consensus model. In contrast, if a substance was within the AD of the first and third models, but not the other two models, it would have a summed weight of 1.4 (0.8 and 0.6, respectively), and it would be within the AD of the consensus model. This majority weight requirement for AD inclusion was applied consistently across all consensus models.

After confirming substance inclusion within the AD, the next step is to determine the prediction result, which is also determined using majority weighting. For predictions, a consensus is reached when most of the models, as determined by their respective weights, predict either a positive (or active for a toxicological endpoint) or negative (inactive) outcome. An example is using the same component model BA values as above (i.e., 0.8, 0.75, 0.65, and 0.6) where all four models returned predictions for a given substance. The consensus prediction is derived by comparing the combined BA values for positive (active) *versus* negative (inactive) predictions. For instance, in the context of predicting ER activity, if two models that predict a substance to bind to ERs have BA values of 0.8 and 0.65, respectively (sum of 1.45), and the models predicting non-binding have BA values of 0.75 and 0.6, respectively (sum of 1.35), the consensus model prediction will be “ER binding” (as 1.45 > 1.35).

In this study, we provided metrics for each individual component model. When combining all the models, we applied the six aforementioned combinatorial methods. Additionally, we conducted tests for every possible combination of these models. In other words, if a specific endpoint had four models (A, B, C, and D), combinations like A and B, A and C, A, B, and C, and so forth were systematically tested, ensuring a comprehensive examination of model combinations. This presented a numerical challenge, as shown in Eq. 7, where the number of consensus models (*N*_*Models*_) depends on both the number of combinatorial methods (*r*) and component models (*n*). The number of combinatorial methods was fixed for this work at 6, and the number of component models varied, depending on the endpoint from 4 to 10. Consequently, each endpoint required an evaluation of between 70 and 6,088 consensus models, composed of three subtypes: 1) the individual component models, which are the single models used to create the consensus models; 2) full-consensus models, which are the combination of all available component models; and 3) all other consensus models, comprising combinations of two or more models up to one less than the total number of available models.

$$N_{Models}=r*\left(2^{n}-1\right)-\left(r-1\right)*n.$$ (7)

The consensus model code and sample inputs, as well as instructions for running the codes, are available on GitHub under the Massachusetts Institute of Technology License (https://github.com/SeanPCollins/ConsensusModels).

### 2.5 Evaluation of consensus model performance

As predictive power and coverage are distinct metrics and are not easily combined, this is a multi-objective optimization problem, which uses different terms and methodologies. One such term is the Pareto front, which represents the set of non-dominated solutions, where improving one objective comes at the cost of performance in another (Ngatchou et al., 2005). In simpler terms, the Pareto front consists of consensus models that offer the best performance across various metrics, regardless of trade-offs. By presenting Pareto fronts, the choice for a trade-off is left to the end user. In this work, when looking at the Pareto fronts, the metrics considered were the coverage and ModP. The ModP was chosen to represent the predictive power as it not only considered a holistic term like the balanced accuracy but also placed a focus on maximizing the number of positives correctly predicted. Here, a best solution is determined by calculating the distance to the ideal solution, namely, 100% coverage and a ModP of 1. The Pareto front is determined by iterating over the consensus models and comparing the ModP and coverage of a given model against all other models. If no other model outperforms all metrics of interest, the model is considered a Pareto front model. The single best Pareto front model is referred to here as the optimal consensus model.

### 2.6 Bootstrapping analysis of Pareto front trends

To assess the statistical significance of the observed trends in our results, bootstrapping techniques were used (Efron and Tibshirani, 1994). Bootstrapping is a computational method that involves iterative resampling of our data. It starts with recording the initial results and then proceeds through multiple rounds of shuffling and resampling. After each shuffling, new results are recorded, creating a distribution of simulated outcomes. The core idea behind bootstrapping is that if the observed results are unrelated to the testing method or influenced by random variations, they should be replicable through a random chance. In this study, bootstrapping was applied to analyze two key aspects: the inclusion of models within the Pareto front and the average number of component models within Pareto consensus models. The component models of all Pareto models and the average number of component models in the Pareto models were tracked. When shuffling, a consistent number of Pareto front models were maintained while randomly assigning Pareto front classifications to the consensus models. By creating a randomized distribution of component model frequencies and the average number of models within the Pareto front, comparisons could be made to determine how often similar or more extreme results occurred. The less often it occurred, the more statistically relevant the result. Here, Pareto front composition trends that were observed less than 5% of the time in the bootstrapping analysis were considered to be statistically significant. Additional information on the bootstrapping analysis is available in Supplementary Materials (Section 3).

## 3 Results

Two performance metrics were considered to assess the models: ModP (Eq. 6) and the coverage. The ModP term was developed based on work previously done by the USEPA, where the predictivity of a model is described. The equation was based on the BA of the model and the absolute difference between its sensitivity and specificity. In this approach, model performance is viewed as optimal if the sensitivity and specificity are as close as possible while maintaining a high BA. Given that the study aims to develop an *in silico* predictive approach that maximizes the use of various available model sources and minimizes false negative predictions to be incorporated in high-throughput screening methods, less emphasis was placed on the difference between the two terms. Instead, emphasis was placed on the absolute value of the sensitivity, as shown in Eq. 6. Applying this focus encourages a high BA, increases sensitivity, and minimizes false negatives. For the intended context of use, this was considered more pragmatic as the *in silico* tier serves to rapidly screen a large number of diverse chemistries and will extend higher confidence that substances with a potential for toxicity will be captured and progress to higher tiers of screening, testing, and assessment, as relevant.

### 3.1 Estrogen receptor activity models

**FIGURE 1:**
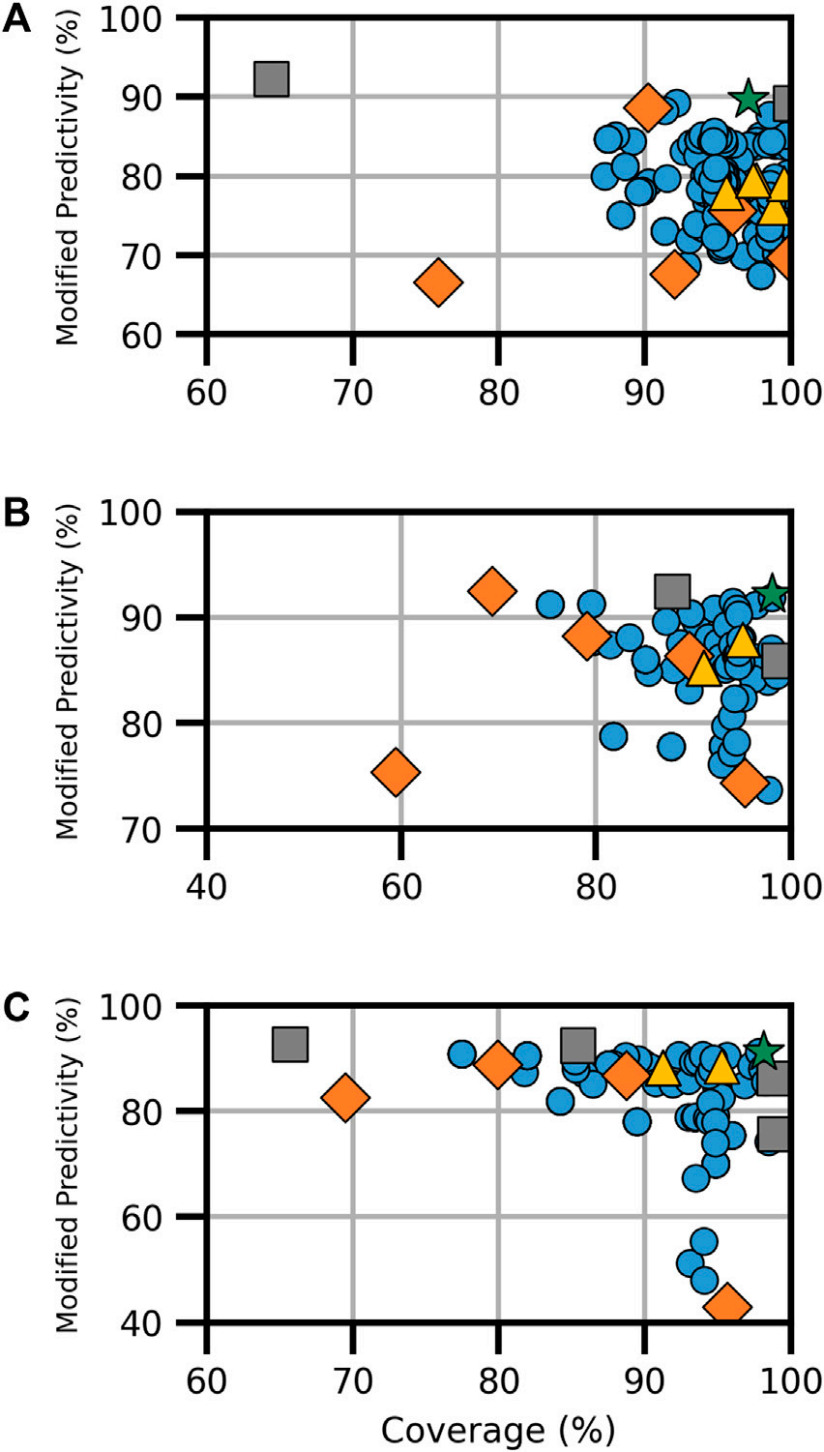
Performance metrics for consensus models developed for ER **(A)** binding, **(B)** agonism, and **(C)** antagonism. Orange diamonds are the component models, yellow triangles are the full-consensus models (all models combined), gray squares are the Pareto front models, the green star is the best performing consensus model, and blue circles are all other consensus models.

For ER activity, six models existed for binding and five each for agonism and antagonism. Each activity type featured the CERAPP consensus model, an HC RF model, and at least one CU model. Binding activity also had the VEGA, ADMET, and ACD models, while agonism and antagonism had two additional CU models (Supplementary Table S4). The combination of available models resulted in a total of 348 models for ER binding and 161 each for ER agonism and antagonism, as shown in Figure 1. The analysis revealed a broad spectrum of performance in terms of coverage and ModP. Notably, HC RF models performed the best among the models examined when considering both ModP and coverage. In contrast, consensus models created using all component models (i.e., full-consensus models) had tightly centered results. In general, when all available models are combined, the performance increases compared to the individual component models, although some increases were moderate. Further testing with the Pareto front models, which constitute the selection of optimal models depending on the degree of the trade-off considered, proved to increase the performance significantly over other consensus and component models (Table 3).

**TABLE 3:** ER-activity optimal Pareto models.

**Endpoint**	**Combination**	**# Models**	**Coverage** **(%)**	**ModP**	**Component model names**
ER binding	Majority	2	97.1	0.897	Health Canada ER binding random forest
					CASE Ultra ER
ER agonist	Majority	2	98.1	0.921	CERAPP ER agonist consensus
					CASE Ultra ER agonist
ER antagonist	Majority	2	98.2	0.912	CERAPP ER antagonist consensus
					CASE Ultra ER antagonist

For ER binding, the Pareto front comprised three consensus models from a pool of 348 assessed models (Figure 1A). Notably, the CU ER model appeared in every Pareto front consensus model, and the component CU model was also a Pareto front solution. In this instance, the CU model had the highest ModP (0.922) but a low coverage at 64.4%. Conversely, the VEGA, ADMET ER, and CERAPP ER consensus models were absent from all ER-binding Pareto front models. The bootstrapping analysis revealed that the chance of any of these models being absent from all Pareto front consensus models was roughly 10% (Supplementary Table S5). When selecting a single consensus model as the best based on the distance to the ideal solution (hereafter referred to as the “optimal consensus model”), the Pareto front model composed of the majority combination of CU ER and HC RF models proved to be the best choice, yielding a ModP of 0.897 and coverage of 97.1% (Table 3).

For ER agonism, among the 161 models (Figure 1B), only three were a part of the Pareto front, and notably, none of the Pareto front models included the CU ER agonist beta model. This finding could potentially be attributed to the limited number of consensus models; however, the bootstrapping analysis revealed that this result (i.e., the CU ER agonist model not being in any Pareto front models) occurred only 8% of the time by random chance (Supplementary Table S6). When assessing the distance to the ideal solution as a singular metric, the “optimal” consensus model was the majority combination of the CERAPP and CU ER agonist models, with a ModP of 0.921 and coverage of 98.1% (Table 3).

ER antagonism (Figure 1C) had a total of 161 models, out of which we identified five Pareto models. The bootstrapping analysis revealed the most notable results for the HC RF model and CU ER antagonist beta model, both of which were only in one of the five Pareto front models. This was observed only approximately 12% of the time by chance (Supplementary Table S7). The “optimal” consensus model was the majority combination of the CERAPP and CU ER antagonist models, with a coverage of 98.2% and ModP of 0.912 (Table 3). The full bootstrapping results for the ER endpoints are presented in Supplementary Tables S5–S7 and Supplementary Figure S1.

### 3.2 Androgen receptor activity models

**FIGURE 2:**
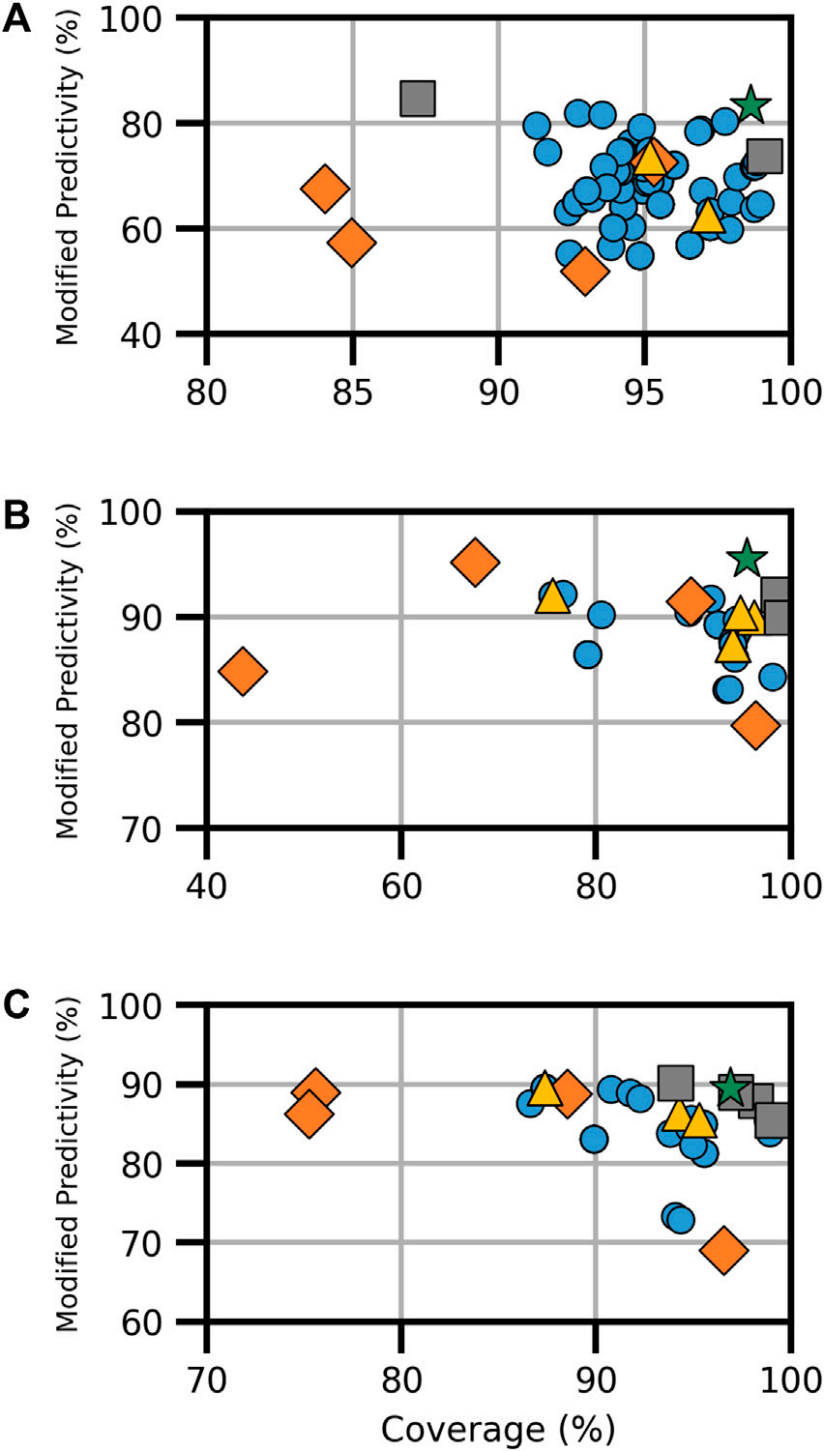
Performance metrics for consensus models developed for AR **(A)** binding, **(B)** agonism, and **(C)** antagonism. Orange diamonds are the component models, yellow triangles are the full-consensus models (all models combined), gray squares are the Pareto front models, the green star is the best performing consensus model, and blue circles are all other consensus models.

For the AR activity models, five models existed for binding and four each for agonism and antagonism. Each AR endpoint featured a CoMPARA consensus model and an HC RF model. Binding activity also had the VEGA, ADMET, and Oasis TIMES models, while agonism and antagonism had two additional CU models (Supplementary Table S8). The combination of available models resulted in a total of 151 models for AR binding and 70 each for AR agonism and antagonism (Figure 2). The current analysis revealed a broad spectrum of performance in terms of coverage and ModP. As with the ER endpoint, the HC RF models performed the best among the component models examined when considering both ModP and coverage, and when all available models were combined, the performance tended to increase compared with individual component models.

Of the 161 AR-binding models evaluated, three formed the Pareto front (Figure 2A). Notably, the HC RF model, as a Pareto front model, achieved the highest ModP at 0.847 but had the lowest coverage of the Pareto models at 87.2%. The bootstrapping results showed the most significant result was that VEGA and Oasis TIMES were not present in any Pareto front models, an event that happened only 8.3% and 7.8% of the time, respectively, by chance (Supplementary Table S9). The optimal consensus model was the majority combination of the HC RF and CoMPARA models, achieving a coverage of 98.6% and a ModP of 0.833 (Table 4).

**TABLE 4:** AR-activity optimal Pareto models.

**Endpoint**	**Combination**	**# Models**	**Coverage** **(%)**	**ModP**	**Component model names**
AR binding	Majority	2	98.6	0.833	CoMPARA AR binding consensus
					Health Canada AR binding random forest
AR agonist	Majority	2	95.5	0.955	Health Canada AR agonist random forest
					CASE Ultra AR agonist MDA
AR antagonist	Majority	2	96.9	0.894	Health Canada AR antagonist random forest
					CASE Ultra AR antagonist HEK

For the AR agonist and AR antagonist endpoints, there was a limited pool of component models, resulting in 70 models in total for each endpoint (Supplementary Table S8). In the AR agonism Pareto front, we identified three solutions (Figure 2B). The bootstrapping analysis showed the only relevant result of the Pareto consensus model compositions was that the CU AR agonist human embryonic kidney (HEK) model appeared in no consensus models, a result shared with only 5.2% of random results (Supplementary Table S10). The “optimal” Pareto model was the majority combination of the HC RF and CU AR agonist for MD Anderson (MDA) cell line (CU AR agonist MDA) models, with a coverage of 95.5% and ModP of 0.955 (Table 4).

The AR antagonism Pareto front comprised five solutions, including the majority combination of all four models (Figure 2C). The CU AR antagonist HEK and HC RF models were the most common component models, appearing in four Pareto solutions. Although this is frequent, it was not sufficient to be considered statistically relevant as the bootstrapping results showed those models appeared in that frequency or higher roughly 35.8% of the time (Supplementary Table S11). Similar to AR agonism, the optimal model in terms of the distance to ideal was the majority combination of the HC RF and CU antagonist HEK models. The resulting consensus model had a coverage of 96.9% and ModP of 0.894 (Table 4). The full bootstrapping results for the AR endpoints are presented in Supplementary Tables S9–S11 and Supplementary Figure S2.

### 3.3 Genotoxicity models

**FIGURE 3:**
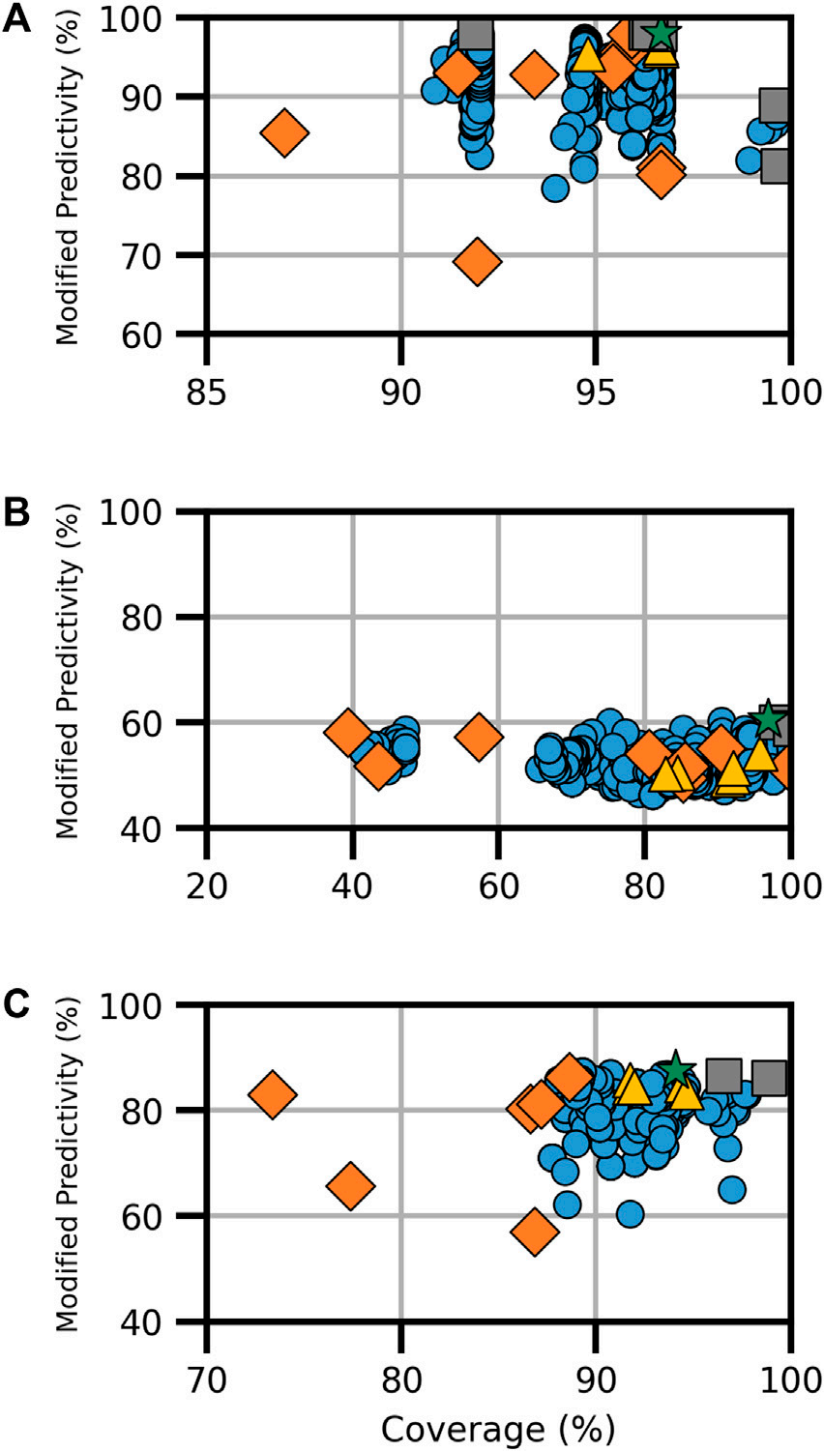
Performance metrics for consensus models developed for genotoxicity, specifically **(A)** bacterial mutation, **(B)** *in vitro* chromosomal aberration, and **(C)** *in vivo* micronucleus. Orange diamonds are the component models, yellow triangles are full-consensus models (all models combined), gray squares are the Pareto front models, the green star is the best performing consensus model, and blue circles are all other consensus models.

In addition to assessing the endocrine-disrupting activity, consensus models were built for genotoxicity endpoints, specifically bacterial mutation, *in vitro* chromosomal aberration, and *in vivo* micronucleus, based on 10, 8, and 6 component models, respectively (Supplementary Table S12). The consensus models for genotoxicity are shown in Figure 3.

The bacterial mutation assay, commonly known as the Ames assay, was predicted using 10 component models, the combination of which resulted in a total of 6,088 assessed models; from these, eight Pareto models were determined (Figure 3A). Notably, the model applier (MA) expert model was the most common component model, observed in seven of the eight Pareto solutions, including on its own where coverage was 96.4% and ModP was 0.982 (Supplementary Table S12). The bootstrapping analysis revealed that this is a statistically relevant finding as the MA expert model was observed at this level or higher only 3.5% of the time by random chance (Supplementary Table S13). Additionally, the MA Ames model was commonly found in Pareto solutions, appearing in five of the eight Pareto models; however, this also occurred 37.2% of the time by chance. Conversely, the ChemTunes (CT) Ames and TIMES Ames models were not in any Pareto front models, a significant result observed only 0.4% of the time in the bootstrapping results. The optimal bacterial mutation consensus model was the majority combination of the HC RF, MA Ames, MA expert, and CU genotoxicity expert models, resulting in a coverage of 96.7% and ModP of 0.980 (Table 5). Although this combination was the best performing model, it only represented a slight improvement compared to the MA expert model alone. Many of the Ames Pareto front models clustered closely based on performance metrics.

**TABLE 5:** Genotoxicity optimal Pareto models.

**Endpoint**	**Combination**	**# Models**	**Coverage** **(%)**	**ModP**	**Component model names**
Bacterial mutagenicity	Majority	4	96.7	0.980	Health Canada AMES random forest
					Model applier Ames
					Model applier expert
					CASE Ultra GT EXPERT
*In vitro* chrom. aberration	Majority	2	96.9	0.605	Health Canada ChromAb random forest
					Model Applier ChroModel Applier CHL
*In vivo* micronucleus (MN)	Majority	4	94.1	0.875	Health Canada *in vivo* random forest
					GT3 MNT MOUSE
					Model Applier MN
					VEGA-MN

Chrom. aberration/Chromab, chromosomal aberration; MNT, micronucleus test.

There were eight component models for the *in vitro* chromosomal aberration endpoint, resulting in 1,490 total models, including three Pareto solutions (Figure 3B). The HC RF model was found in all three Pareto solutions, while four component models, namely, the MA ChromAb Chinese hamster ovary (CHO) model, CT-ChromAb, and both the CU GT2 Chrom CHO and CHL, were found in none. These results were not statistically relevant according to the bootstrapping analysis, appearing in approximately 12% of the random samples (Supplementary Table S14). The “optimal” Pareto solution was the majority combination of the HC RF and MA ChromAb Chinese hamster lung (CHL) models with a coverage of 96.9% and ModP of 0.605 (Table 5).

The final genotoxicity endpoint examined was the *in vivo* micronucleus, which used six component models to create 348 total models for assessment, including three Pareto models (Figure 3C). Due to the limited number of Pareto front models and the available number of component models, no statistically significant trends in Pareto model composition were observed. The HC RF and MA micronucleus (MN) models were in all three Pareto solutions, and the TIMES MN model was not a component of any of the Pareto solutions. The exclusion of the TIMES MN model was the most notable as it was observed in only 9.9% of the bootstrapping results (Supplementary Table S15). The optimal consensus model was the majority combination of the HC RF, GT3 MNT mouse, MA MN, and VEGA MN models, which had a coverage of 94.1% and ModP of 0.875 (Table 5). The full bootstrapping results for the genotoxicity endpoint are presented in Supplementary Tables S13–S15, Supplementary Figure S3.

### 3.4 Summary of Pareto model composition analysis

In addition to the composition of the Pareto front models, the total number of models used in the Pareto front was also analyzed (Supplementary Table S16). The major conclusion drawn from this analysis was that the Pareto front models showed a preference for a low number of models. For example, for the ER-binding endpoint, the three actual Pareto front models averaged 1.66 models per consensus model, while the average from the bootstrapping results was 3.33 (Supplementary Figure S1). When analyzing the bootstrapping results where the mean number of models was 1.66 or less, this was only observed 0.32% of the time by chance, making the low number of models used a statistically relevant result. This trend was also observed for ER antagonist (mean 2 models) (Supplementary Figure S1), AR binding (mean 1.66 models) (Supplementary Figure S2), bacterial mutagenicity (mean 2.875 models) (Supplementary Figure S3), and *in vitro* chromosomal aberration (mean 2 models) (Supplementary Figure S3) models. The bootstrapping results showed that the mean number of models was that low, or lower, only 2.37%, 1.52%, 0%, and 0.18% of the time, respectively. The only endpoint where the average number of models was higher than its respective bootstrapping result was AR antagonism, which had a mean of 2.6 compared to the bootstrap result of 2.4 (Supplementary Figure S2). Finally, the values for bootstrapping across all endpoints yielded an average of 3.22 models or 55.7% of all available models. The actual results had an average of 2.37 models or 40.9% of all available models, with the bootstrapping results reaching those levels, or lower, 19.22% and 6.43% of the times, respectively. In this instance, there is a difference in the bootstrapping results between the number and percentage of models due to the difference in the number of available models for each endpoint. For that reason, the better comparison to make is using the percentage of available models, meaning the 40.9% result, which the bootstrapping analysis demonstrated only reached that percentage composition or lower 6.43% of the time by chance.

### 3.5 Model coverage for the Canadian Domestic Substances List

**FIGURE 4:**
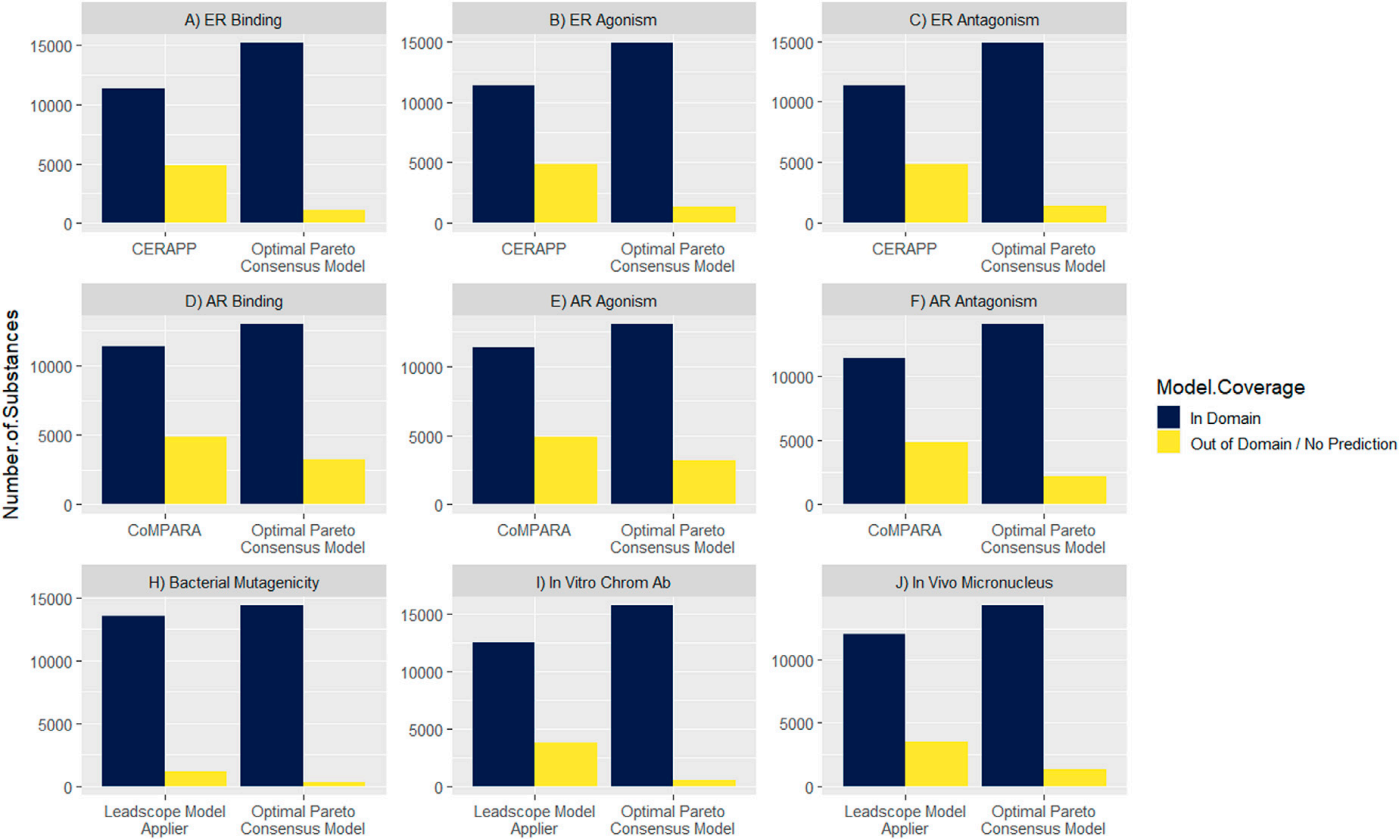
Comparison of model coverage between optimal Pareto consensus models vs. a selection of individual component models across Canada’s Domestic Substances List (DSL).

Maximizing the model coverage across the DSL is an important outcome for the work presented here. Canada’s DSL contains approximately 28,000 substances, of which approximately 16,000 substances are amenable to modeling (i.e., have a defined or representative chemical structure). The DSL coverage of the optimal Pareto consensus models was compared with a selection of the individual component models (Figure 4). For the ER-related endpoints, the CERAPP model coverage was 70% across endpoints, while the optimal Pareto consensus models improved the coverage range from 91% to 93%. A similar improvement was achieved for the AR-related endpoints; the CoMPARA model coverage was 70% across models, which was improved to 79%–86% with the application of the optimal Pareto consensus models. For genotoxicity endpoints, the Leadscope model coverage ranged from 77% to 92%, which was improved to 92%–98% with the optimal Pareto consensus models.

## 4 Discussion

The application of consensus models for toxicity predictions can play an important role in regulatory toxicology, offering a powerful tool to screen and prioritize substances for further testing and assessment. Here, it has been demonstrated that the use of consensus models enables a high-throughput and comprehensive evaluation of potential toxicity that can be carried out across multiple endpoints. Improvements in predictivity and chemical space coverage were demonstrated using training sets that spanned a large chemical space. The use of *in silico* predictive models for prioritization and assessment aligns with increasing reliance on NAM-based, or “next-generation risk assessment” (NGRA), approaches in toxicology (Madden et al., 2020; Cronin et al., 2022) and leverages successful models like CERAPP for ER activity (Mansouri et al., 2016) and CoMPARA for AR activity (Mansouri et al., 2020). The application of the Pareto front approach to identify optimal consensus models, as shown here, offers a robust and rapid means to enhance predictions from *in silico* models while maximizing chemical space coverage.

Existing datasets originally compiled by the USEPA for CERAPP and CoMPARA (Mansouri et al., 2016; Mansouri et al., 2020), as well as the Leadscope SAR Genetox Database (Leadscope, 2019), were leveraged to extend the utility of established (Q)SAR models. In addition to evaluating the combination of all available models using a variety of different combinatorial rules, every possible combination of models was also tested. While the genotoxicity endpoints had a relatively large number of component models available for each endpoint (i.e., mean of eight), there were fewer ER and AR component models available from the same developers (i.e., a mean of five and four, respectively). The larger number of models available for genotoxicity assessment reflects the fact that it has a long history of being a critical component in the regulatory assessment of chemicals and pharmaceuticals, and *in silico* predictive models offer a cost-effective, time-efficient, NAM-based option for identifying potential genotoxic compounds early on.

In this work, six different combinatorial methodologies and the Pareto front approach were used to evaluate consensus models and demonstrate utility as a flexible and adaptable approach that could be applied across nine toxicological endpoints. A previous study reported a consensus modeling strategy using majority voting and Bayes consensus with discrete probability on AR binding, agonism, and antagonism, considering several publicly reported (Q)SAR models (Valsecchi et al., 2020). This study included several commercial models for ER and AR binding in addition to public domain models. In another study, the authors used various modeling approaches including classic machine learning, normal deep learning, and multitask deep learning to construct *in silico* models to predict ER assay outcomes for 18 ToxCast and Tox21 assays, using a training set of >7,500 chemicals. The results of this study showed that no single algorithm consistently outperformed others across the 18 endpoints, while consensus models formed by averaging predictions from individual models exhibited similar or higher predictivity (Ciallella et al., 2021). Likewise, in this study, it was found that the optimal consensus model performed better than any individual component model across all nine endpoints. In a related study for genotoxicity models, the Naïve Bayes algorithm was used as the aggregating function to combine (Q)SAR models, such as EPA TEST and VEGA, and the structural alerts from the QSAR Toolbox to identify the model with the highest balanced accuracy and sensitivity (Pradeep et al., 2021). While the Naïve Bayes aggregation focuses on probabilistic estimates and assumes feature independence, the unique attribute of Pareto front consensus models is their ability to incorporate the predictions from several diverse models using a variety of combinatorial approaches and identify a single optimal prediction while accounting for overall performance trade-offs. The added value of this methodology has been clearly demonstrated for assessing large inventories of structurally and functionally diverse chemicals, allowing for the integration of information from various models to resolve data conflicts and provide a more reliable outcome while also expanding the domain of applicability.

Using this approach, consensus models for ER activity, AR activity, and genotoxicity were developed that demonstrated improvements in both chemical space coverage and predictivity compared to the individual component models. The endpoints with the highest performing consensus models were the ER endpoints. The coverage across all 3 ER endpoints was >97% and ModP was ≥0.9. The consensus models for the AR endpoints were also quite high performing, with coverage >95% and ModP ranging from 0.833 to 0.955. Regarding the genotoxicity endpoints, models for both the bacterial mutation and *in vivo* micronucleus endpoints also performed very well (coverages of 96.7% and 94.1% and ModPs of 0.980 and 0.875, respectively); however, a notable exception was the *in vitro* chromosomal aberration endpoint. For this endpoint, the “optimal” Pareto solution had a high coverage (96.9%) but a ModP of only 0.604. Across all Pareto solutions for *in vitro* chromosomal aberration, ModP had an upper limit of 0.606. This limitation is attributed to a strong and significant negative correlation between sensitivity and specificity across all models. For this endpoint, plotting its sensitivity as a function of specificity yields a slope of approximately −1 and an *R* ^2^ of 0.837, meaning that improving one parameter leads to a decrease in the other. This challenging trade-off between sensitivity and specificity complicates efforts to enhance ModP, which relies on the BA, the average of both sensitivity and specificity.

A bootstrapping analysis was used to analyze trends in the component models that were included in the Pareto front consensus models, as well as the total number of models. For each endpoint, an analysis was performed to see whether there was a statistical relevance to each model appearing in the Pareto fronts, which would suggest that those models are more likely to yield enhanced performance. A few trends were noted; however, these largely failed to meet the level of statistical significance, likely due to the low number of Pareto front consensus models and/or component models. When looking across all endpoints, it was possible to find trends in the number of models used to construct each consensus model and its relationship to inclusion in the Pareto front. The combination of all available models did not result in the highest performance when considering predictive power (i.e., ModP) and chemical space coverage, consistent with previous results (Kulkarni and Barton-Maclaren, 2014; Abdelaziz et al., 2016; Pradeep et al., 2021). These combinations, while generally improving upon the individual model performances, did not outperform consensus models constructed from a more limited selection of models, a trend that held true across all endpoints. This implies that some models within the full consensus models could introduce noise, lowering their overall performance. The bootstrapping analysis revealed that across all endpoints, the mean percentage of models that were in the Pareto front was only 40.9%, a result that approached statistical significance as it would be expected to happen by chance only 6.4% of the time. Finally, it was notable that although six different combinatorial methods were used for each possible combination of models, the optimal consensus models for all nine endpoints were by a majority combination. This means that each of the component models included in the optimal consensus models contributed a similar weight to the consensus outcome. A key takeaway from this work is that the use of a workflow such as the one presented in this study, where every combination of available models is tested using multiple combinatorial methods, is necessary if the aim is to find the best possible consensus model for predictions. This process needs to be performed on each available endpoint, with the results analyzed to determine the best consensus model for a particular use.

Finally, we demonstrated the application of this approach to improve screening of a large chemical inventory of structurally diverse compounds. As mentioned, consensus models are well-suited to high-throughput screening as they provide a single outcome per endpoint, avoiding the need to address data conflicts while maximizing the utility for chemical screening and prioritization. In addition, one of the advantages of developing consensus models is that they expand the chemical space coverage. Approximately 16,000 substances exist on Canada’s DSL that are amenable to *in silico* modeling. Across all nine endpoints, an improvement in chemical space coverage was observed when applying the optimal Pareto consensus models developed here compared with a component model. The expansion of the chemical space coverage allows for screening of more chemicals on the DSL for potential hazard, which translates to a better ability to identify those chemicals that show potential to impact the health of people living in Canada.

In summary, a methodology was developed to build *in silico* consensus models, allowing the combination of multiple *in silico* (Q)SAR models to provide a single prediction for a given toxicological endpoint. Using high-quality datasets for nine toxicological endpoints and 4–10 *in silico* component models per endpoint, we applied six combinatorial methods. The models that formed the Pareto front were optimized for both coverage and ModP. It should be noted that the Pareto front models outperformed the full-consensus models, showing the importance of comprehensive screening of potential consensus models to ensure optimal solutions. Overall, consensus models demonstrated improved performance compared to individual component models, particularly in terms of the coverage of chemical space and predictive power. The application of a distinctive Pareto front-based consensus approach resulted in robust models with improved coverage and predictive power. These enhancements made these consensus models ideally suited for application to large inventories of data-poor chemicals, such as Canada’s DSL, to enhance the overall outcome reliability of (Q)SAR models. Combining multiple predictions broadens knowledge and reliability, mitigating contradictory information effects, and extends the applicability domain in the chemical space. These consensus models are developed to be integrated into a larger multi-tier NAM-based framework to prioritize chemicals for further investigation and support the transition to a non-animal approach to risk assessment in Canada.

## Data Availability

The original contributions presented in the study are included in the article/Supplementary Material; further inquiries can be directed to the corresponding author.
