# Supplementary material for: Development and application of consensus in silico models for advancing high-throughput toxicological predictions
Source: Front Pharmacol. 2024 Jan 25;15:1307905. doi: 10.3389/fphar.2024.1307905 (PMC10850302; doi:10.3389/fphar.2024.1307905)
Supplement: Supplementary file 1 [file Table1.DOCX]

**Supplementary Materials for Development and Application of Consensus in silico Models for Advancing High-Throughput Toxicological Predictions**

Sean P. Collins^1*^, Brandon, Mailloux^1^, Sunil Kulkarni^1^, Matthew Gagné^1^ Alexandra S. Long^1^, Tara S. Barton-Maclaren^1^

^1^Exsisting Substances Risk Assessment Bureau, Health Environments and Consumer Safety Branch, Health Canada Ottawa, ON, K1A 0K9

Contents

[1. Details of the validation datasets 3](#_Toc152089308)

[2. Details of the k-Nearest Neighbour (kNN) approach for consensus models 3](#_Toc152089309)

[3. Statistical Analysis Using Bootstrapping 4](#_Toc152089310)

[4. Tables 5](#_Toc152089311)

[5. Figures 11](#_Toc152089312)

[6. References 13](#_Toc152089313)

# Details of the validation datasets

The datasets came from three different sources, with the Collaborative Estrogen Receptor Activity Prediction Project (CERAPP)(Mansouri et al., 2016) providing estrogen receptor (ER) data, Collaborative Modeling Prediction of Androgen Reception Activity (CoMPARA)(Mansouri et al., 2020) for androgen receptor (AR) data, and the genotoxicity data came from the Leadscope SAR Genetox database.(Leadscope, 2019)

The CERAPP and CoMPARA datasets were created using similar methods. For this work, the ER and AR datasets used were known as the ‘Evaluation’ datasets from their original works. The reason for using the Evaluation datasets is due to their size, allowing for a larger chemical space coverage. The datasets were constructed using in vitro data that was collected from a wide variety of sources and using custom adverse outcome pathways related to specific assays and mathematical equations, they were able to derive dose-response curves. From those curves, the area under the curves (AUCs) was derived which could be related to the potential of a substance for the specific endpoints.

For the Genetox data, the data was first extracted from the Leadscope SAR Genetox database and then filtered. First, only data points that were either positive or negative in the validation sets for each assay. Leadscope was able to make overall calls for the substances' overall assays available for a specific endpoint, such as bacterial mutagenicity. To keep the results conservative, only substances where all available assays agreed with the overall call were kept. This would mean that if a substance had an overall call of negative, and one of the assays had a positive call it was removed. To be specific, the bacterial mutation was based upon available strain data in Salmonella and E. coli, with and/or without metabolic activation. For the in vitro chromosomal aberration, the calls were based on available assay results in rodent cell lines and/or human blood cells. Finally, for in vivo micronucleus, the overall calls were based upon available data in rodents and/or humans.

# Details of the k-Nearest Neighbour (kNN) approach for consensus models

The k-Nearest Neighbor (kNN) combinatorial approach for consensus models begins by utilizing the fingerprint values of a known substances database, which are associated with well-defined chemical properties. The fingerprints were a combination of multiple freely available structural fingerprints and some calculated physical-chemical properties for all chemicals. The structural fingerprints included were PubChem fingerprints (Kim et al., 2019), MACCS fingerprints, and the FP2, FP3, and FP4 fingerprints from OpenBabel (O’Boyle et al., 2011), as well as fifteen physical chemistry properties calculated using OpenBabel. Most structural fingerprints are a series of 1’s and 0’s indicating the presence or absence of a chemical feature or substructure, such as if the substance has four or more carbon atoms or contains an aldehyde. The physical-chemical properties included molecular weight, number of atoms and bonds, and predicted values such as melting point and logP. These descriptors were chosen as they cover various substructures and properties. In total, 2456 descriptors were calculated for each substance and combined to create a single fingerprint. Before calculating any distances, for each dataset descriptor columns with no variance or were highly correlated to another descriptor column (noted with a correlation coefficient greater than 0.95) were removed.

On the pruned fingerprints, dimensionality reduction was done by using Factor Analysis (FA) which can better handle binary and continuous variables in the same combination with no pre-processing. In FA, the standard metric to describe how well it fits is the noise variance, which is the amount of variance not explained in the fit components of FA. For example, in one dataset the total variance was 21649, and when a single component FA was performed, the noise variance was 16920. If the difference is taken, 4729, that would mean the component explained 21.8% of the variance. For this work a threshold of 98% was chosen for explained variance, although a minimum was set to have 3 components. Using these requirements, the number of components ranged from 3 to 9, depending on the database.

The FA transformed data for each substance could act as dimensional coordinated for the substance, which was used to calculate the twelve nearest neighbours. For those twelve nearest neighbours, the average distance was calculated and used as the weight for that substance for that model. For instance, if a substance X in model A has a kNN average distance of 5, and for model B it was 7, it would give a higher weighting to the model A for substance X than for model B, as there are more known substances closer to substance X in the applicability domain for model A. To calculate the weights each model applied, the similarity value (S) for the D_mean­_, average distance, (shown in equation S1) was used. This would favour models that a substance was very close to the known values (therefore low distance) over those where the substance was much different than other values (high distance).

|  | $S=\frac{1}{D_{mean}+1}$ | (S1) |
| --- | --- | --- |

# Statistical Analysis Using Bootstrapping

Bootstrapping is a computational technique where results are randomly shuffled and then resampled to create a random distribution of the results. By repeating the process a sufficient number of times, the distribution is sufficient to conclude if the results from the original test are statistically significant or not. This is used in this work by resampling which models are in the Pareto front, while keeping the number of models in the Pareto front constant. The average number of models in the Pareto consensus models were examined, as well as which models appeared in the Pareto consensus models. Ten-thousand shuffles were performed to create the distribution and then determined how many random samples were as extreme or more extreme than the real results.

# Tables

Table S1: Information on (Q)SAR model training set sources

| Model name | Source | Nature of Model |
| --- | --- | --- |
| ACD_ER | US FDA, METI and other souces. | Statistical |
| CU_ER | Mansouri et al. 2016 | Statistical |
| CU_ER_Agonist | Mansouri et al. 2016 | Statistical |
| CU_ER_Agonist_alpha | ToxCast/Tox21 | Statistical |
| CU_ER_Agonist_beta | ToxCast/Tox21 | Statistical |
| CU_AR_Agonist | ToxCast/Tox21 | Statistical |
| CU_AR_Antagonist_alpha | ToxCast/Tox21 | Statistical |
| CU_AR_Antagonist_beta | ToxCast/Tox21 | Statistical |
| CU_AR_Agonist_HEK | ToxCast/Tox21 | Statistical |
| CU_AR_Agonist_MDA | ToxCast/Tox21 | Statistical |
| CU_AR_Antagonist_HEK | ToxCast/Tox21 | Statistical |
| CU_AR_Antagonist_MDA | ToxCast/Tox21 | Statistical |
| TIMES_AR | National Center for Toxicology Research (Fang et al., 2003) | Categorical fragments based |
| VEGA_AR | COMPARA | Statistical |
| VEGA_ER | CERAPP | Statistical |
| ADMET_AR | DSSTox | Statistical |
| ADMET_ER | DSSTox | Statistical |
| MA_Ames | US FDA | Statistical |
| MA_Expert | Rules-based | Expert rule-based |
| TIMES_Ames | Mix of proprietary and public databases; CCRIS, Toxnet, US EPA, NCI, P&G, etc. | Hybrid metabolic |
| CU_GT1_BMUT | Public databases | Statistical |
| CU_GT_EXPERT | Rules-based | Expert rule-based |
| ACD_Ames | CCRIS, GENE-TOX | Statistical |
| ADMET_Ames | NIHS Japan | Statistical |
| VEGA_Ames | Public databases | Statistical |
| CT-Ames | US FDA CFSAN  US FDA Drugs@FDA  EU SCCS, SCCP, SCCNFP  EU ECHA REACH (Registered Substance Database)  US NTP (National Toxicology Program)  Published literature with detailed experimental information | Statistical |
| MA_ChromAb CHL | US FDA | Statistical |
| MA_ChromAb CHO | US FDA | Statistical |
| TIMES_ChrAb with S9 | Sofuni, T., Ed. 1998. | Hybrid metabolic |
| GT2_CHROM_CHL | US FDA CDER | Statistical |
| GT2_CHROM_CHO | US FDA CDER | Statistical |
| CT-ChrAb | US FDA CFSAN  US FDA Drugs@FDA  EU SCCS, SCCP, SCCNFP  EU ECHA REACH (Registered Substance Database)  US NTP (National Toxicology Program)  Published literature with detailed experimental information | Statistical |
| GT3_MNT_MOUSE | US FDA CDER | Statistical |
| MA_MN | US FDA | Statistical |
| TIMES_MN | Various literature sources | Hybrid metabolic |
| CT-MN | US FDA CFSAN  US FDA Drugs@FDA  EU SCCS, SCCP, SCCNFP  EU ECHA REACH (Registered Substance Database)  Published literature with detailed experimental information | Statistical |
| VEGA-MN | Public databases | Statistical |

Table S2. (Q)SAR models used for each endpoint

| Endpoint | US EPA Models (Mansouri et al., 2016, 2020) | CaseUltra (Saiakhov et al., 2013) | Oasis TIMES (Todorov et al., 2011) | Health Canada RF (Collins and Barton-Maclaren, 2022) | VEGA (Satyanarayan et al., 2016) | ADMET Predictor (Simulations-Plus, 2020) | ACD (Advanced Chemistry Development, 2019) | Chem Tunes | Model Applier |
| --- | --- | --- | --- | --- | --- | --- | --- | --- | --- |
| ER Binding | CERAPP | CU_ER |  | HC RF | VEGA_ER | ADMET_ET | ACD_ER |  |  |
| ER Agonism | CERAPP | CU_ER_Agonist  CU_ER_Agonist_Alpha  CU_ER_Agonist_Beta |  | HC RF |  |  |  |  |  |
| ER Antagonism | CERAPP | CU_AR_Agonist  CU_AR_Antagonist_Alpha  CU_AR_Antagonist_Beta |  | HC RF |  |  |  |  |  |
| AR Binding | CoMPARA |  | TIMES_AR | HC RF | VEGA_AR | ADMET_AR |  |  |  |
| AR Agonism | CoMPARA | CU_AR_Agonist_HEK  CU_AR_Agonist_MDA |  | HC RF |  |  |  |  |  |
| AR Antagonism | CoMPARA | CU_AR_Antagonist_HEK  CU_AR_Antagonist_MDA |  | HC RF |  |  |  |  |  |
| AMES |  | CU_GT_Expert  CU_GT1_BMUT | TIME_Ames with S9 | HC RF | VEGA_Ames | ADMET_Ames | ACD_Ames | CT-Ames | MA_Ames  MA_Expert |
| in vitro ChromAb |  | CU_GT2_CHROM_CHL  CU_GT2_CHROM_CHO | TIMES_ChrAb with S9 | HC RF |  |  |  | CT-ChrAb | MA_ChromAb CHL  MA_ChromAb CHO |
| in vivo micronucleus |  | CU_G3T_MNT_Mouse | TIMES_MN with S9 | HC RF | VEGA-MN |  |  | CT-MN | MA_MN |

Table S3: QMRF links* for various (Q)SAR models

| Name of model | QMRF link |
| --- | --- |
| VEGA | <https://www.vegahub.eu/portfolio-item/vega-qsar-models-qrmf/> |
| ADMET Predictor | <https://qsardb.org/repository/handle/10967/246> |
| CaseUltra | <https://jeodpp.jrc.ec.europa.eu/ftp/jrc-opendata/EURL-ECVAM/datasets/QSARDB/LATEST/PDF/_qmrf_protocol_Q13-410-0070_document.pdf> |
| ACD | <https://jeodpp.jrc.ec.europa.eu/ftp/jrc-opendata/EURL-ECVAM/datasets/QSARDB/LATEST/PDF/_qmrf_protocol_Q15-410-0003_document.pdf> |

**Weblinks not available for all models. Most QMRFs are available as pdfs within the software interface.*

Table S4 – ER-activity component model performance

| **Endpoint** | **Model Name** | **Coverage** | **ModP** |
| --- | --- | --- | --- |
| ER binding | CERAPP ER Binding Consensus | 96.0% | 0.756 |
|  | Health Canada ER Binding Random Forest | 90.2% | 0.886 |
|  | CaseUltra ER | 64.4% | 0.923 |
|  | ACD ER | 100.0% | 0.696 |
|  | ADMET ER | 92.1% | 0.675 |
|  | VEGA ER | 75.9% | 0.665 |
| ER agonist | CERAPP ER Agonist Consensus | 95.3% | 0.743 |
|  | Health Canada ER Agonist Random Forest | 89.6% | 0.863 |
|  | CaseUltra ER AGONIST | 69.4% | 0.924 |
|  | CaseUltra ER AGONIST ALPHA | 79.1% | 0.882 |
|  | CaseUltra ER AGONIST BETA | 59.4% | 0.753 |
| ER antagonist | CERAPP ER Antagonist Consensus | 95.7% | 0.429 |
|  | Health Canada ER Antagonist Random Forest | 88.8% | 0.868 |
|  | CaseUltra ER ANTAGONIST | 65.7% | 0.927 |
|  | CaseUltra ER ANTAGONIST ALPHA | 79.9% | 0.888 |
|  | CaseUltra ER ANTAGONIST BETA | 69.5% | 0.825 |

Table S5. Results of composition of Pareto front, and its comparison to bootstrapping results for ER binding.

| Model | Frequency observed in Pareto Front | Times that Frequency or further from median was observed in bootstrapping results (%) |
| --- | --- | --- |
| CaseUltra ER | 3 | 15.07 |
| VEGA ER | 0 | 9.48 |
| HC ER Binding RF | 1 | 44.12 |
| ADMET ER | 0 | 9.67 |
| CERAPP ER Binding | 0 | 9.68 |
| ACD ER | 1 | 44.56 |

Table S6. Results of composition of Pareto front, and its comparison to bootstrapping results for ER agonism.

| Model | Frequency observed in Pareto Front | Times that Frequency or further from median was observed in bootstrapping results (%) |
| --- | --- | --- |
| CaseUltra ER Agonist | 2 | 50 |
| CaseUltra ER Agonist Alpha | 2 | 50 |
| CaseUltra ER Agonist Beta | 0 | 8.1 |
| HC ER Agonist RF | 1 | 40.36 |
| CERAPP ER Agonist | 2 | 50 |

Table S7. Results of composition of Pareto front, and its comparison to bootstrapping results for ER antagonism.

| Model | Frequency observed in Pareto Front | Times that Frequency or further from median was observed in bootstrapping results (%) |
| --- | --- | --- |
| CaseUltra ER Antagonist | 3 | 50 |
| CaseUltra ER Antagonist Alpha | 2 | 37.15 |
| CaseUltra ER Antagonist Beta | 1 | 11.63 |
| HC ER Antagonist RF | 1 | 10.74 |
| CERAPP ER Antagonist | 3 | 50 |

Table S8 – AR activity component model performance

| **Endpoint** | **Model Name** | **Coverage** | **ModP** |
| --- | --- | --- | --- |
| AR binding | CoMPARA AR Binding Consensus | 95.3% | 0.726 |
|  | Health Canada AR Binding Random Forest | 87.2% | 0.847 |
|  | ADMET | 84.1% | 0.675 |
|  | VEGA | 85.0% | 0.573 |
|  | Oasis TIMES | 93.0% | 0.518 |
| AR agonist | CoMPARA AR Agonist Consensus | 96.4% | 0.797 |
|  | Health Canada AR Agonist Random Forest | 89.8% | 0.914 |
|  | CaseUltra AR AGONIST HEK | 43.7% | 0.848 |
|  | CaseUltra AR AGONIST MDA | 67.6% | 0.951 |
| AR antagonist | CoMPARA AR Antagonist Consensus | 96.6% | 0.690 |
|  | Health Canada AR Antagonist Random Forest | 88.5% | 0.887 |
|  | CaseUltra AR ANTAGONIST HEK | 75.6% | 0.889 |
|  | CaseUltra AR ANTAGONIST MDA | 75.3% | 0.862 |

Table S9. Results of composition of Pareto front, and its comparison to bootstrapping results for AR binding.

| Model | Frequency observed in Pareto Front | Times that Frequency or further from median was observed in bootstrapping results (%) |
| --- | --- | --- |
| ADMET AR | 1 | 40.52 |
| VEGA AR | 0 | 7.87 |
| Oasis TIMES AR | 0 | 7.87 |
| HC AR Binding RF | 2 | 50 |
| CERAPP AR Binding | 2 | 50 |

Table S10. Results of composition of Pareto front, and its comparison to bootstrapping results for AR agonism.

| Model | Frequency observed in Pareto Front | Times that Frequency or further from median was observed in bootstrapping results (%) |
| --- | --- | --- |
| CaseUltra AR Agonist HEK | 0 | 5.46 |
| CaseUltra AR Agonist MDA | 2 | 50 |
| HC AR Agonist RF | 2 | 50 |
| CoMPARA AR Agonist RF | 2 | 50 |

Table S11. Results of composition of Pareto front, and its comparison to bootstrapping results for AR antagonism.

| Model | Frequency observed in Pareto Front | Times that Frequency or further from median was observed in bootstrapping results (%) |
| --- | --- | --- |
| CaseUltra AR Antagonist HEK | 4 | 35.11 |
| CaseUltra AR Antagonist MDA | 3 | 50 |
| HC AR Antagonist RF | 4 | 34.73 |
| CoMPARA AR Antagonist RF | 2 | 28.75 |

Table S12 – Genotoxicity component model performance

| **Endpoint** | **Model Name** | **Coverage** | **ModP** |
| --- | --- | --- | --- |
| Bacterial mutagenicity | Health Canada AMES Random Forest | 92.0% | 0.691 |
|  | Model Applier Ames | 95.9% | 0.979 |
|  | Model Applier Expert | 96.4% | 0.982 |
|  | TIMES Ames | 96.7% | 0.801 |
|  | CaseUltra GT1 BMUT | 91.5% | 0.930 |
|  | CaseUltra GT EXPERT | 95.7% | 0.956 |
|  | ACD Ames | 93.4% | 0.928 |
|  | ADMET Ames | 96.7% | 0.810 |
|  | VEGA Ames | 95.4% | 0.935 |
|  | Chem Tunes-Ames | 87.0% | 0.854 |
| In vitro chromosomal aberration (Chrom Ab) | Health Canada Chrom Ab Random Forest | 85.5% | 0.516 |
|  | Model Applier ChroModel Applierb CHL | 80.6% | 0.539 |
|  | Model Applier ChroModel Applierb CHO | 85.3% | 0.494 |
|  | TIMES ChrAb with S9 | 90.5% | 0.550 |
|  | GT2 CHROM CHL | 39.4% | 0.580 |
|  | GT2 CHROM CHO | 43.5% | 0.517 |
|  | Chrom Aberr | 99.9% | 0.515 |
|  | Chem Tunes-ChrAb | 57.3% | 0.572 |
| In vivo micronucleus (MN) | Health Canada in vivo Random Forest | 87.2% | 0.812 |
|  | GT3 MNT MOUSE | 73.4% | 0.830 |
|  | Model Applier MN | 88.6% | 0.863 |
|  | TIMES MN | 86.9% | 0.570 |
|  | Chem Tunes-MN | 77.4% | 0.656 |
|  | VEGA-MN | 86.6% | 0.803 |

Table S13. Results of composition of Pareto front, and its comparison to bootstrapping results for bacterial mutagenicity.

| Model | Frequency observed in Pareto Front | Times that Frequency or further from median was observed in bootstrapping results (%) |
| --- | --- | --- |
| MA Ames | 5 | 37.21 |
| MA Expert | 7 | 3.43 |
| CaseUltra GT1 BMUT | 1 | 3.39 |
| CaseUltra GT Expert | 2 | 14.09 |
| CT-Ames | 0 | 0.35 |
| ACD Ames | 2 | 13.69 |
| VEGA Ames | 2 | 13.78 |
| TIMES Ames | 0 | 0.35 |
| ADMET Ames | 1 | 3.58 |
| HC Ames RF | 3 | 36.9 |

Table S14. Results of composition of Pareto front, and its comparison to bootstrapping results for in vitro chromosomal aberration.

| Model | Frequency observed in Pareto Front | Times that Frequency or further from median was observed in bootstrapping results (%) |
| --- | --- | --- |
| GT2 Chrom CHL | 0 | 11.24 |
| GT2 Chrom CHO | 0 | 11.89 |
| CT-ChromAb | 0 | 11.9 |
| MA ChromAb CHL | 1 | 47.71 |
| MA ChromAb CHO | 0 | 11.68 |
| TIMES ChrAb with S9 | 1 | 48.75 |
| ChromAberr | 1 | 47.61 |
| HC ChromAb RF | 3 | 13.89 |

Table S15. Results of composition of Pareto front, and its comparison to bootstrapping results for in vivo micronucleus.

| Model | Frequency observed in Pareto Front | Times that Frequency or further from median was observed in bootstrapping results (%) |
| --- | --- | --- |
| GT3 MNT Mouse | 2 | 50 |
| CT-MN | 1 | 43.49 |
| VEGA MN | 1 | 45.45 |
| TIMES MN | 0 | 9.88 |
| HC in vivo MN RF | 3 | 15.05 |
| MA MN | 3 | 15.37 |

Table S16. Bootstrap results compared against observed results as well as percentage of bootstrap results as far or further from the median value.

| Endpoint | Mean Number of Models | Bootstrap Mean Number of Models | Mean Percentage of Models (%) | Bootstrap Percentage of Models (%) | Bootstrap Samples as far or Further from Median (%) |
| --- | --- | --- | --- | --- | --- |
| ER Binding | 1.66 | 3.33 | 27.7 | 55.6 | 0.32 |
| ER Agonist | 2.33 | 2.66 | 46.6 | 53.3 | 26.91 |
| ER Antagonist | 2 | 2.8 | 40 | 56 | 2.37 |
| AR Binding | 1.66 | 2.66 | 33.3 | 53.3 | 1.52 |
| AR Agonist | 2 | 2.33 | 50 | 58.3 | 24.58 |
| AR Antagonist | 2.6 | 2.4 | 65 | 60 | 43.6 |
| Bacterial Mutagenicity | 2.875 | 5 | 28.75 | 50 | 0 |
| In vitro Chromosomal Aberration | 2 | 4 | 25 | 50 | 0.18 |
| In vivo Micronucleus | 3.33 | 3.33 | 55.6 | 55.6 | 50 |
| Total | 2.37 | 3.22 | 40.9 | 55.7 | 19.22/6.43^*^ |

^*^Results are for number of models and percentage of models, respectively. The values differ due as each endpoint uses a different number of models.

# Figures


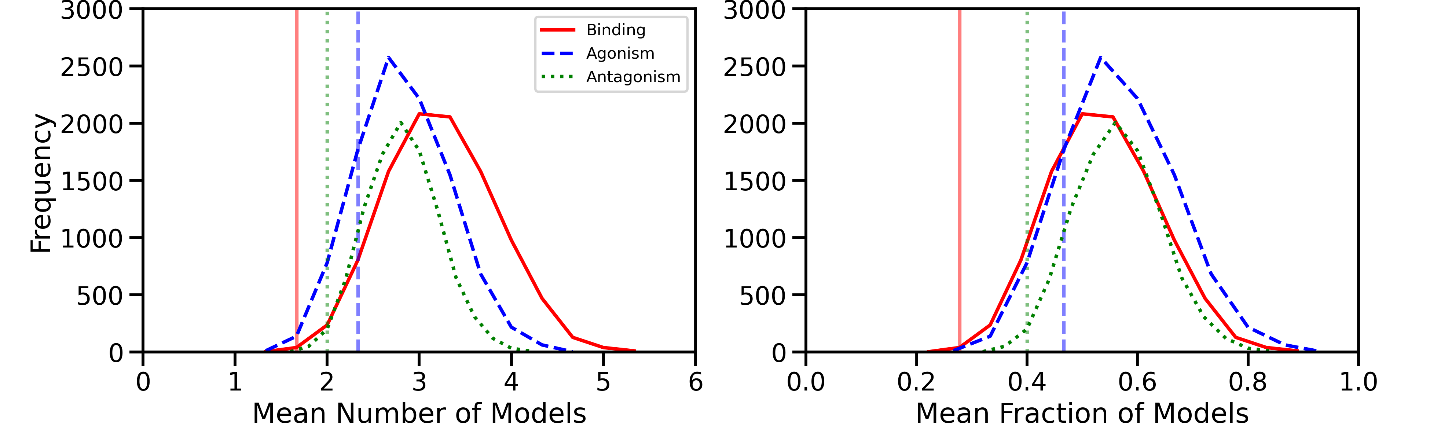


Figure S1. Random distribution created by bootstrapping for ER activity. Results are shown for absolute number of models on the left, and fraction of the total models on the right. Red is for ER binding, blue for ER agonism, and green is for ER antagonism. The corresponding vertical line is the results found in this work.


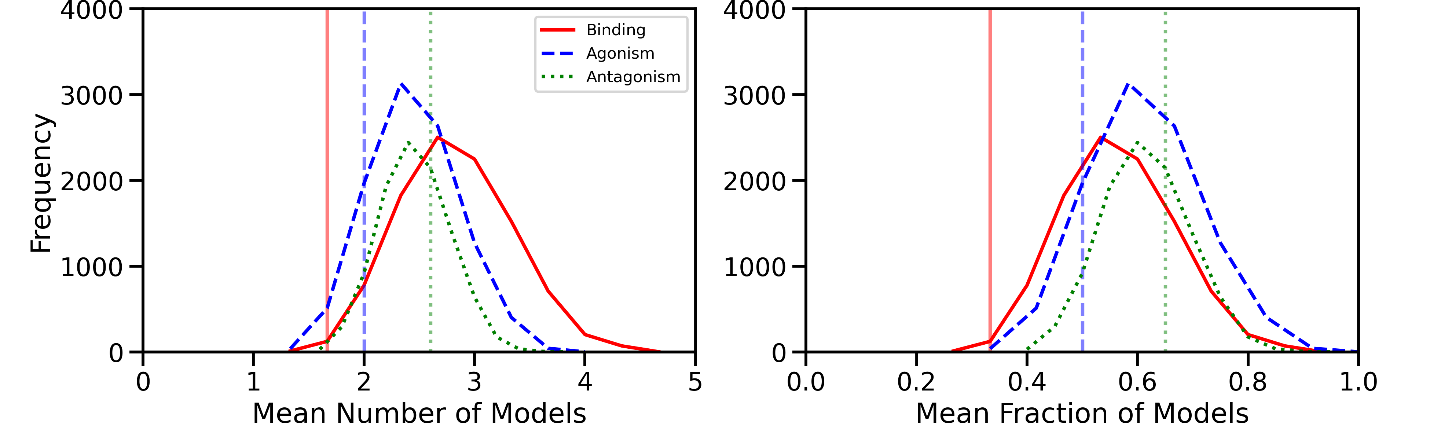


Figure S2. Random distribution created by bootstrapping for AR activity. Results are shown for absolute number of models on the left, and fraction of the total models on the right. Red is for AR binding, blue for AR agonism, and green is for AR antagonism. The corresponding vertical line is the results found in this work.


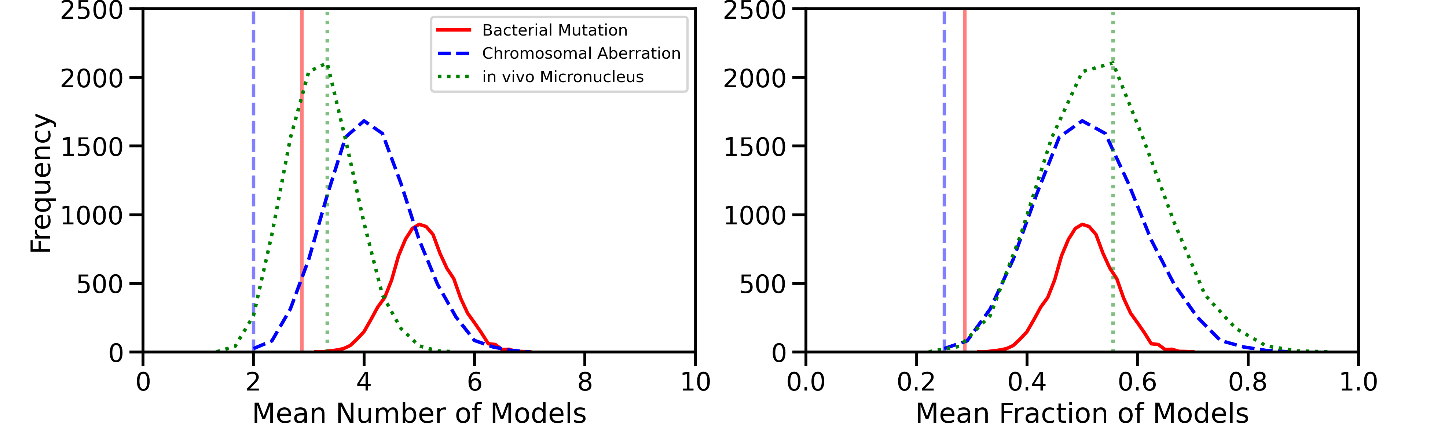


Figure S3. Random distribution created by bootstrapping for Genotoxicity. Results are shown for absolute number of models on the left, and fraction of the total models on the right. Red is for bacterial mutagenicity, blue for in vitro chromosomal aberration, and green is for in vivo micronucleus. The corresponding vertical line is the results found in this work.


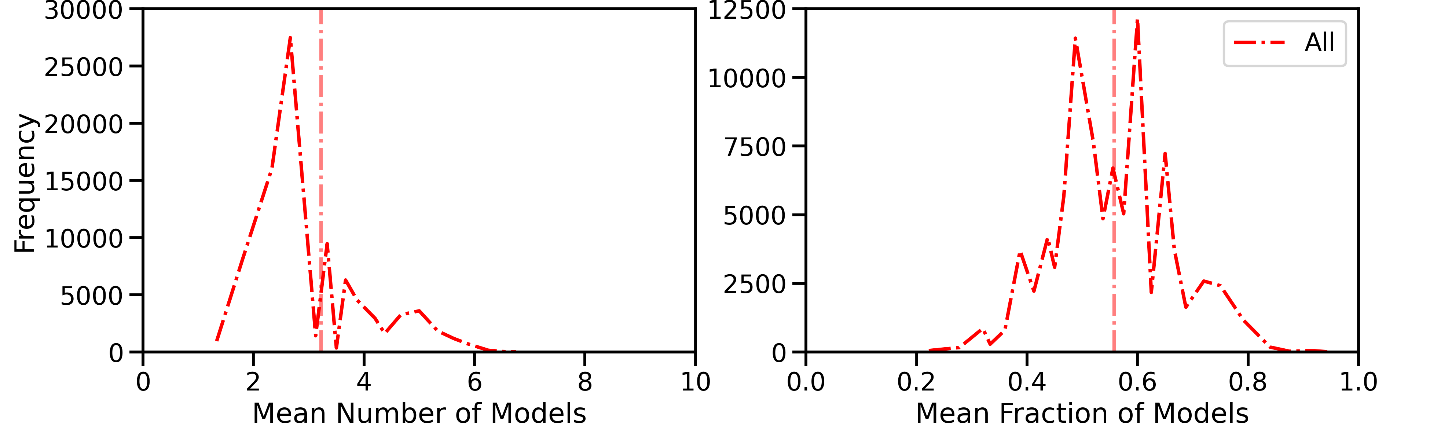


Figure S4. Random distribution created by bootstrapping for all endpoints. Results are shown for absolute number of models on the left, and fraction of the total models on the right. The vertical line is the results found in this work.

# References

Advanced Chemistry Development (2019). ACD Percepta version 2019. Available at: www.acdlabs.com.

Collins, S. P., and Barton-Maclaren, T. S. (2022). Novel machine learning models to predict endocrine disruption activity for high-throughput chemical screening. *Front. Toxicol.* 4, 117. doi:10.3389/ftox.2022.981928.

Kim, S., Chen, J., Cheng, T., Gindulyte, A., He, J., He, S., et al. (2019). PubChem 2019 update: improved access to chemical data. *Nucleic Acids Res.* 47, D1102–D1109. doi:10.1093/nar/gky1033.

Leadscope (2019). SAR Genetox Database: Leadscope. Available at: http://www.leadscope.com/toxicity_databases/ [Accessed February 25, 2022].

Mansouri, K., Abdelaziz, A., Rybacka, A., Roncaglioni, A., Tropsha, A., Varnek, A., et al. (2016). CERAPP: Collaborative Estrogen Receptor Activity Prediction Project. *Environ. Health Perspect.* 124, 1023–1033. doi:10.1289/ehp.1510267.

Mansouri, K., Kleinstreuer, N., Abdelaziz, A. M., Alberga, D., Alves, V. M., Andersson, P. L., et al. (2020). CoMPARA: Collaborative Modeling Project for Androgen Receptor Activity. *Environ. Health Perspect.* 128, 027002. doi:10.1289/EHP5580.

O’Boyle, N. M., Banck, M., James, C. A., Morley, C., Vandermeersch, T., and Hutchison, G. R. (2011). Open Babel: An open chemical toolbox. *J. Cheminform.* 3, 33. doi:10.1186/1758-2946-3-33.

Saiakhov, R., Chakravarti, S., and Klopman, G. (2013). Effectiveness of CASE Ultra Expert System in Evaluating Adverse Effects of Drugs. *Mol. Inform.* 32, 87–97. doi:10.1002/minf.201200081.

Satyanarayan, A., Russell, R., Hoffswell, J., and Heer, J. (2016). Reactive Vega: A Streaming Dataflow Architecture for Declarative Interactive Visualization. *IEEE Trans. Vis. Comput. Graph.* 22, 659–668. doi:10.1109/TVCG.2015.2467091.

Simulations-Plus (2020). ADMET Predictor.

Todorov, M., Mombelli, E., Aït-Aïssa, S., and Mekenyan, O. (2011). Androgen receptor binding affinity: a QSAR evaluation. *SAR QSAR Environ. Res.* 22, 265–291. doi:10.1080/1062936X.2011.569508.
